# Signal mining and analysis of influencing factors for adverse events of Nivolumab and Cetuximab in the treatment of head and neck cancer based on the US FAERS database

**DOI:** 10.3389/fimmu.2025.1658535

**Published:** 2025-10-29

**Authors:** Yi Tang, Yongchuan She, Danping Chen, Yibo Zhou, Zhai Liu, Zhi Chen, Jun Wan, Yu Ren

**Affiliations:** Changsha Hospital of Traditional Chinese Medicine (Changsha Eighth Hospital), Changsha, China

**Keywords:** head and neck cancer (HNC), adverse event (ADE), Nivolumab, Cetuximab, FDA Adverse Event Reporting System (FAERS)

## Abstract

**Background:**

No prior research has directly compared adverse drug event (ADE) profiles of Nivolumab and Cetuximab in head and neck cancer (HNC) using the US FDA Adverse Event Reporting System (FAERS). The present study aims to evaluate ADE signal characteristics of both agents to inform clinical decision-making.

**Methods:**

Data extracted from FAERS included patient baseline characteristics, which were summarized in a baseline table. Disproportionality analysis with reporting odds ratio (ROR) and Bayesian confidence propagation neural network (BCPNN) was applied to identify signals at the system organ class (SOC) and preferred term (PT) levels.

**Results:**

For Nivolumab, three significant SOC-level signals were identified—benign/malignant tumors (including cysts/polyps), hepatobiliary disorders, and endocrine abnormalities. At the PT level, 58 effective signals were observed, with immune-related events such as thyroid dysfunction being particularly frequent. For Cetuximab, 40 effective PT-level signals were detected, dominated by dermatologic toxicity (rash) and metabolic abnormalities (hypomagnesemia). Comparative analysis revealed marked differences between the two drugs: Nivolumab was more strongly associated with immune-mediated reactions, whereas Cetuximab was characterized by cutaneous and metabolic toxicity.

**Conclusions:**

This study represents the first FAERS-based assessment of ADE risk differences between Nivolumab and Cetuximab in HNC, offering valuable evidence for clinical monitoring and drug selection. As signal detection reflects statistical correlation rather than causality, confirmatory clinical validation remains necessary. Integration of real-world evidence with prospective clinical trials will be essential to enhance drug safety evaluation systems.

## 1 Introduction

Head and neck cancer (HNC) includes malignant tumors arising in the oral cavity, pharynx, and larynx, with squamous cell carcinoma representing the predominant histological subtype (1).The disease severely impairs quality of life, and the complexity of local anatomy often renders therapeutic outcomes suboptimal (2).Its onset is closely associated with long-term carcinogenic exposure, including tobacco use and alcohol consumption (3).Current therapeutic modalities consist of surgery, radiotherapy, and chemotherapy (4).However, these approaches are frequently accompanied by high recurrence rates and treatment-related toxicity. The need for more effective therapeutic strategies has therefore become evident (5).In recent years, immunotherapy has emerged as a promising approach in HNC management, particularly with the use of immune checkpoint inhibitors (ICIs) such as Nivolumab and targeted agents like Cetuximab. Both agents have demonstrated measurable benefits in prolonging survival and improving quality of life, yet adverse events remain a significant concern (6).

Importantly, Nivolumab and Cetuximab represent pharmacologically distinct classes (7, 8). Nivolumab, as an immune checkpoint inhibitor (ICI), exerts its effect by enhancing host immune activity against tumor cells (7, 9), while Cetuximab acts as a molecular targeted therapy, directly interfering with tumor cell proliferation through inhibition of the epidermal growth factor receptor (EGFR) signaling cascade (10). The divergence in their mechanisms of action results in distinct toxicity profiles: ICIs predominantly induce immune-related adverse events (irAEs), whereas EGFR inhibitors are associated with dermatologic toxicity and metabolic disturbances (11, 12). A systematic comparison of the safety profiles of these two agents is thus scientifically justified and clinically necessary to support the development of individualized treatment strategies.

Nivolumab and cetuximab demonstrate targeted therapeutic efficacy in HNC and HNSCC. Nivolumab significantly extends survival in patients with platinum-resistant HNSCC (13). Cetuximab, a molecularly targeted agent for head and neck cancers, exerts variable effects when combined with chemotherapy or radiotherapy, and prior investigations indicate potential benefits from such combinations (14). Both agents, however, are associated with adverse reactions: nivolumab commonly induces pruritus and rash, while cetuximab can lead to mucositis, diarrhea, and hypomagnesemia (15). Furthermore, the incidence of severe adverse events related to cetuximab use has been reported to be higher outside the United States. These toxicities substantially impair patient quality of life and therefore necessitate careful monitoring and management throughout therapy.

The Food and Drug Administration Adverse Drug Event Spontaneous Reporting System (FAERS) serves as a repository for adverse event reports concerning drugs and biologics regulated by the FDA (16). This system is indispensable for post-marketing pharmacovigilance, enabling early detection of safety concerns, guiding regulatory interventions, and supporting evidence-based clinical decision-making (17). Accordingly, the present study aims to evaluate the occurrence and determinants of adverse events during treatment of head and neck cancer with Nivolumab and Cetuximab, with the ultimate goal of informing clinical strategies and advancing individualized therapeutic approaches.

## 2 Materials and methods

### 2.1 Data extraction and filtering

The original dataset was derived from the US FAERS database (https://fis.fda.gov/extensions/FPD-QDE-FAERS/FPD-QDE-FAERS.html), which compiled spontaneous adverse drug event (ADE) reports submitted by healthcare providers, patients, and other sources. The database, updated quarterly and publicly accessible at no cost, is stored in ASC or XML format and frequently applied in signal detection for marketed drugs. By March 1, all ADE reports related to HNC recorded since the database’s inception were retrieved. The search employed the keyword “Head and Neck Cancer,” with recognition that the FAERS system distinguishes between “Head and Neck Cancer” and “Head and Neck Squamous Cell Carcinoma.” For subsequent analyses, both categories were consolidated and treated uniformly as HNC.

To evaluate the clinical safety profile of Nivolumab and Cetuximab, targeted searches with the keywords “Nivolumab” and “Cetuximab” were performed, capturing ADE reports from Q1–2004 through Q4 2024. The identified reports were systematically organized and annotated according to the preferred terms (PT) and system organ classes (SOC) defined by Medical Dictionary for Regulatory Activities (MedDRA). SOCs were arranged by etiology (e.g., infectious and parasitic diseases), anatomical site (e.g., gastrointestinal disorders), or purpose (e.g., surgical and medical procedures). Each PT denotes a discrete medical concept including symptoms, diagnoses, indications, examinations, or clinical procedures. While every PT is mapped to at least one SOC, multiple SOC associations are possible depending on context.

From the first quarter of 2004 through the fourth quarter of 2024, ADE reports of Nivolumab and Cetuximab were collected and subjected to systematic data cleaning. Reports were excluded according to the following criteria: (1) ADE IDs associated with Nivolumab or Cetuximab for HNC and HNSCC that had been officially deleted, duplicated, or missing in the FDA database; (2) reports containing inconsistent demographic variables, including gender, age, weight, or country of occurrence. Subsequent data refinement applied further criteria: (1) duplicate entries across demographic (DEMO), drug usage (DRUG), adverse reaction (REAC), patient outcome (OUCT), report source (RPSR), and treatment duration (THER) datasets were removed to enhance reliability; (2) drug-related adverse event IDs for HNC flagged as deleted, duplicated, or missing by the FDA were excluded; (3) reports lacking information on gender, age, severity outcomes, or onset days were also discarded. After cleaning, 3840 reports remained, comprising 2038 for Nivolumab and 1802 for Cetuximab (**Supplementary Table 1**).

### 2.2 Data mining

Signal detection for ADEs of Nivolumab and Cetuximab was conducted using disproportionality analysis. This method evaluates the relative difference between observed frequencies of drug–event pairs and expected background frequencies through a fourfold contingency table. Within this framework, the reporting odds ratio (ROR) and proportional reporting ratio (PRR) serve as standard indices, whereas the Bayesian Confidence Propagation Neural Network (BCPNN) approach emphasizes the Information Component (IC).

### 2.3 Head-to-head analysis of ADE signals between Nivolumab and Cetuximab

To delineate differences in ADE risk at both the SOC and PT levels between Nivolumab and Cetuximab in HNC, a two-way cross disproportionality analysis was performed in addition to single-drug signal mining. ROR with 95% CI served as the principal statistical measure, and a positive signal was defined by two conditions: at least three ADE reports and the lower bound of the ROR 95% confidence interval (CI) > 1. Forest plots were constructed using the forestploter package (Version 1.1.1, https://CRAN.R-project.org/package=forestploter) in R (Version 4.3.1) to display comparative risks of the two agents across PT levels.

### 2.4 Statistical analysis

All statistical procedures were executed in R software (Version 4.3.1), including data cleaning, signal detection, and visualization. Categorical variables, including gender, age, and reporter type, were expressed as frequencies and percentages (n, %). Between-group comparisons, such as baseline characteristics across drug cohorts, were assessed by Chi-square test; when expected frequencies were < 5, Fisher’s exact test was applied. Two-tailed tests were consistently adopted, and statistical significance was set at *P <* 0.05. Data processing and supplementary analyses were additionally supported by MYSQL 8.0, Navicat Premium 15, and GraphPad Prism 8.

## 3 Results

### 3.1 The essential characteristics of HNC drug therapy

To characterize HNC drug treatment, 3,840 FAERS reports concerning Cetuximab and Nivolumab from Q1–2004 to Q4–2024 were examined, comprising 1,802 cases for Cetuximab and 2,038 for Nivolumab.

#### 3.1.1 Nivolumab

As summarized in **Table 1**, the distribution of Nivolumab-related reports revealed several patterns. Male patients accounted for a higher proportion than females, indicating a greater frequency of adverse reactions in men. Individuals weighing between 50 and 100 kg constituted the majority, while age groups 18–64.9 years and 65–85 years represent substantial shares of the cohort. Reports submitted by MDs predominated among reporter types, and Japan emerges as a major reporting country.

**Table 1:** Basic characteristics of ADE related to Nivolumab treatment for HNC in the FAERS database.

	Level	Overall
		(N=2038)
SEX	F	409 (20.1%)
	M	1474(72.3%)
	Missing	155 (7.6%)
WT	<50 kg	164 (8.0%)
	>100 kg	18 (0.9%)
	50 ˜ 100 kg	574 (28.2%)
	Missing	1282(62.9%)
AGE	<18 years	9 (0.4%)
	>85 years	17 (0.8%)
	18 ˜ 64.9 years	823 (40.4%)
	65 ˜ 85 years	732 (35.9%)
	Missing	457 (22.4%)
OCCP_COD	CN	476 (23.4%)
	HP	406 (19.9%)
	LW	1 (0.0%)
	MD	709 (34.8%)
	OT	380 (18.6%)
	PH	65 (3.2%)
	Missing	1 (0.0%)
REPORTER_COUNTRY	ALGERIA	1 (0.0%)
	ARGENTINA	3 (0.1%)
	AUSTRALIA	11 (0.5%)
	BELGIUM	16 (0.8%)
	BRAZIL	8 (0.4%)
	CANADA	21 (1.0%)
	CHILE	1 (0.0%)
	CHINA	87 (4.3%)
	COLOMBIA	16 (0.8%)
	CROATIA	2 (0.1%)
	CZECHIA	1 (0.0%)
	DENMARK	1 (0.0%)
	EGYPT	4 (0.2%)
	FINLAND	1 (0.0%)
	FRANCE	280 (13.7%)
	GERMANY	156 (7.7%)
	HONG KONG	4 (0.2%)
	HUNGARY	8 (0.4%)
	INDIA	290 (14.2%)
	IRELAND	7 (0.3%)
	ISRAEL	1 (0.0%)
	ITALY	22 (1.1%)
	JAPAN	607 (29.8%)
	KOREA, SOUTH	1 (0.0%)
	KUWAIT	1 (0.0%)
	LEBANON	15 (0.7%)
	LITHUANIA	1 (0.0%)
	LUXEMBOURG	1 (0.0%)
	MEXICO	26 (1.3%)
	NETHERLANDS	2 (0.1%)
	NEW ZEALAND	1 (0.0%)
	NORWAY	26 (1.3%)
	POLAND	8 (0.4%)
	PORTUGAL	2 (0.1%)
	PUERTO RICO	1 (0.0%)
	ROMANIA	2 (0.1%)
	RUSSIA	1 (0.0%)
	SAUDI ARABIA	3 (0.1%)
	SPAIN	26 (1.3%)
	SWEDEN	1 (0.0%)
	SWITZERLAND	2 (0.1%)
	TAIWAN	4 (0.2%)
	THAILAND	6 (0.3%)
	UNITED ARAB EMIRATES	1 (0.0%)
	UNITED KINGDOM	28 (1.4%)
	UNITED STATES	330 (16.2%)
	URUGUAY	1 (0.0%)

#### 3.1.2 Cetuximab

The results (**Table 2**) revealed that males receiving Cetuximab accounted for a higher proportion than females, with a correspondingly greater incidence of adverse reactions. Individuals weighing 50–100 kg constituted the majority of cases, and adults aged 18–64.9 years as well as 65–85 years were the most represented age groups. Reports submitted by consumers (CN) predominated among the reporter categories, while Germany contributes a large share of the reports.

**Table 2:** Basic characteristics of ADE related to Cetuximab treatment for HNC in the FAERS database.

X.	Overall
	(N=1802)
SEX	
F	261 (14.5%)
M	1080 (59.9%)
Missing	461 (25.6%)
WT	
<50 kg	73 (4.1%)
>100 kg	22 (1.2%)
50 ˜ 100 kg	400 (22.2%)
Missing	1307 (72.5%)
AGE	
<18岁	1 (0.1%)
>85岁	9 (0.5%)
18 ˜ 64.9岁	581 (32.2%)
65 ˜ 85岁	507 (28.1%)
Missing	704 (39.1%)
OCCP_COD	
CN	988 (54.8%)
HP	6 (0.3%)
MD	190 (10.5%)
OT	538 (29.9%)
PH	67 (3.7%)
RN	3 (0.2%)
Missing	10 (0.6%)
REPORTER_COUNTRY	
ARGENTINA	22 (1.2%)
AUSTRALIA	10 (0.6%)
AUSTRIA	1 (0.1%)
BELGIUM	9 (0.5%)
BRAZIL	7 (0.4%)
CANADA	8 (0.4%)
CHINA	1 (0.1%)
COLOMBIA	46 (2.6%)
COSTA RICA	1 (0.1%)
COUNTRY NOT SPECIFIED	2 (0.1%)
DOMINICAN REPUBLIC	2 (0.1%)
ECUADOR	2 (0.1%)
FRANCE	42 (2.3%)
GERMANY	811 (45.0%)
GREECE	6 (0.3%)
GUATEMALA	1 (0.1%)
HONDURAS	7 (0.4%)
HUNGARY	1 (0.1%)
INDIA	92 (5.1%)
ITALY	11 (0.6%)
JAPAN	190 (10.5%)
LITHUANIA	2 (0.1%)
MEXICO	12 (0.7%)
NETHERLANDS	1 (0.1%)
NICARAGUA	1 (0.1%)
PUERTO RICO	1 (0.1%)
ROMANIA	1 (0.1%)
RUSSIA	3 (0.2%)
SPAIN	19 (1.1%)
SWITZERLAND	2 (0.1%)
TAIWAN	5 (0.3%)
TAIWAN, PROVINCE OF CHINA	1 (0.1%)
THAILAND	1 (0.1%)
TRINIDAD AND TOBAGO	1 (0.1%)
TURKEY	1 (0.1%)
UNITED KINGDOM	11 (0.6%)
UNITED STATES	468 (26.0%)

Overall, analysis of FAERS data from Q1–2004 to Q4–2024 on HNC treatment with Nivolumab and Cetuximab indicates consistent patterns: male patients are more frequently affected by adverse reactions, patients weighing 50–100 kg appear most common, and adults aged 18–64.9 years and 65–85 years are the leading age groups. Distinctions between the two drugs primarily involve the composition of reporter types and country-specific distribution.

### 3.2 The trend of time changes in the treatment of HNC

The temporal distribution of ADE reports for Nivolumab and Cetuximab in HNC was examined using FAERS data from Q1–2004 to Q4 2024, with visualization performed in R (“ggplot2”).

**Figure 1:**
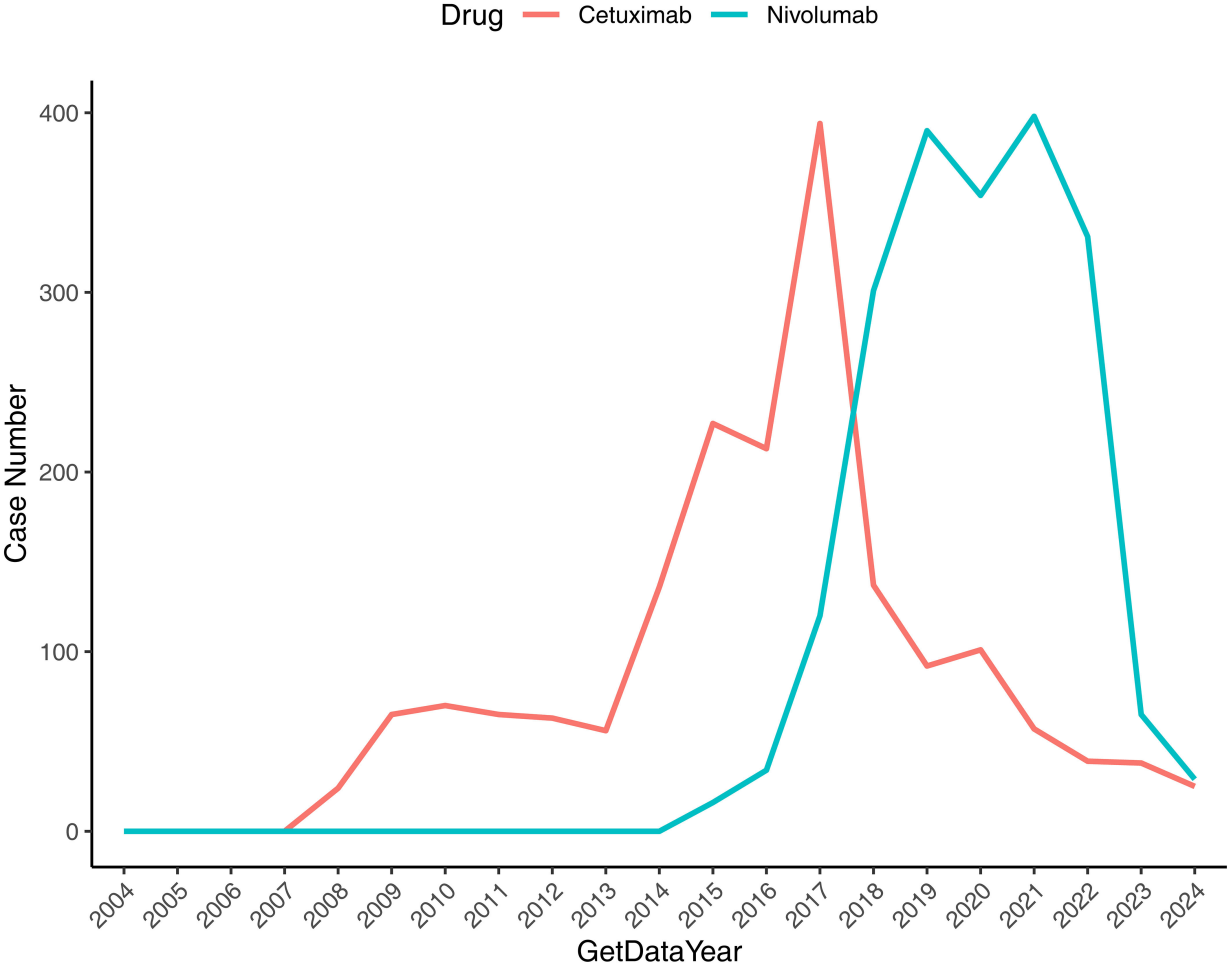
Temporal distribution of reported ADEs associated with drug therapy for HNC. The x-axis denotes the reporting year, and the y-axis indicates the number of drug-related reports.

As shown in **Figure 1**, ADE reports associated with Nivolumab in HNC remained minimal from 2004 to 2016, followed by a sharp increase between 2016 and 2021, although a temporary decline appeared in 2019–2020. From 2021 to 2024, the reporting frequency declined progressively. In contrast, Cetuximab-related ADEs exhibited a steady increase from 2004 until 2017, with a short-lived decline in 2015–2016, and subsequently displayed an overall downward trajectory through 2024.

### 3.3 Analysis of ADE in SOC-level drug treatment for HNC

The SOC-level distribution of ADEs linked to Nivolumab and Cetuximab in HNC was analyzed using FAERS data covering Q1–2004 to Q4 2024. Signal detection was performed through ROR, PRR, and BCPNN analyses, with the criteria for valid signals defined as: ≥ 3 case reports, lower 95% CI of ROR > 1, PRR ≥ 2, χ2 ≥ 4, and IC025 > 0. This approach enabled systematic identification of SOC-level ADE profiles in the context of HNC drug therapy.

#### 3.3.1 Nivolumab

Analysis revealed 25 SOC categories associated with ADEs during Nivolumab therapy for HNC. Among them, NEOPLASMS BENIGN, MALIGNANT AND UNSPECIFIED (including cysts and polyps), HEPATOBILIARY DISORDERS, and ENDOCRINE DISORDERS met the criteria for valid signals (**Table 3**). Red markings in the table denote categories fulfilling all predefined thresholds.

**Table 3:** Signal strength of Nivolumab related to HNC in the FAERS database (SOC level).

soc_name	Case number	ROR (95%Cl)	PRR	χ^2^	IC(IC025)
RESPIRATORY, THORACIC AND MEDIASTINAL DISORDERS	416	1.07 ( 0.95 - 1.2 )	1.06	1.35	0.07 ( -0.09 )
METABOLISM AND NUTRITION DISORDERS	303	0.74 ( 0.65 - 0.84 )	0.76	21.08	-0.32 ( -0.5 )
CARDIAC DISORDERS	131	1.09 ( 0.89 - 1.33 )	1.09	0.71	0.09 ( -0.19 )
GENERAL DISORDERS AND ADMINISTRATION SITE CONDITIONS	901	1.33 ( 1.22 - 1.44 )	1.27	44.27	0.26 ( 0.15 )
INJURY, POISONING AND PROCEDURAL COMPLICATIONS	306	0.98 ( 0.86 - 1.11 )	0.98	0.14	-0.03 ( -0.21 )
INFECTIONS AND INFESTATIONS	460	1.08 ( 0.97 - 1.21 )	1.08	2.01	0.08 ( -0.07 )
NEOPLASMS BENIGN, MALIGNANT AND UNSPECIFIED (INCL CYSTS AND POLYPS)	501	2.48 ( 2.21 - 2.8 )	2.34	241.73	0.85 ( 0.69 )
INVESTIGATIONS	251	0.56 ( 0.49 - 0.64 )	0.58	69.53	-0.64 ( -0.83 )
NERVOUS SYSTEM DISORDERS	257	1.05 ( 0.91 - 1.21 )	1.05	0.4	0.05 ( -0.15 )
EYE DISORDERS	49	1.81 ( 1.28 - 2.56 )	1.8	11.52	0.61 ( 0.13 )
SKIN AND SUBCUTANEOUS TISSUE DISORDERS	234	0.67 ( 0.58 - 0.77 )	0.68	30.86	-0.45 ( -0.65 )
GASTROINTESTINAL DISORDERS	508	0.65 ( 0.58 - 0.71 )	0.68	73.42	-0.45 ( -0.59 )
VASCULAR DISORDERS	83	0.57 ( 0.45 - 0.72 )	0.58	22.77	-0.65 ( -0.99 )
HEPATOBILIARY DISORDERS	162	3.63 ( 2.91 - 4.53 )	3.55	147.67	1.17 ( 0.89 )
MUSCULOSKELETAL AND CONNECTIVE TISSUE DISORDERS	173	2.03 ( 1.67 - 2.45 )	1.99	55.11	0.7 ( 0.45 )
PSYCHIATRIC DISORDERS	84	0.98 ( 0.76 - 1.25 )	0.98	0.04	-0.03 ( -0.37 )
ENDOCRINE DISORDERS	143	5.5 ( 4.22 - 7.16 )	5.37	199.98	1.43 ( 1.13 )
BLOOD AND LYMPHATIC SYSTEM DISORDERS	155	0.42 ( 0.36 - 0.5 )	0.44	104.2	-0.99 ( -1.24 )
SOCIAL CIRCUMSTANCES	3	0.6 ( 0.18 - 2.06 )	0.6	0.66	-0.59 ( -2.17 )
EAR AND LABYRINTH DISORDERS	22	1.64 ( 0.98 - 2.73 )	1.64	3.69	0.52 ( -0.18 )
SURGICAL AND MEDICAL PROCEDURES	13	0.49 ( 0.27 - 0.87 )	0.49	6.12	-0.86 ( -1.68 )
RENAL AND URINARY DISORDERS	110	1.55 ( 1.23 - 1.94 )	1.54	14.39	0.45 ( 0.14 )
PRODUCT ISSUES	8	1.09 ( 0.49 - 2.43 )	1.09	0.05	0.1 ( -0.98 )
IMMUNE SYSTEM DISORDERS	29	0.44 ( 0.3 - 0.64 )	0.44	18.65	-0.99 ( -1.55 )
REPRODUCTIVE SYSTEM AND BREAST DISORDERS	7	3 ( 1.09 - 8.26 )	2.99	4.96	1.04 ( -0.21 )

#### 3.3.2 Cetuximab

A total of 26 SOC categories were identified in relation to Cetuximab treatment of HNC; however, none produced valid ADE signals (**Table 4**).

**Table 4:** Signal strength of Cetuximab related to HNC in the FAERS database (SOC level).

soc_name	Case number	OR (95%Cl)	PRR	χ^2^	IC(IC025)
METABOLISM AND NUTRITION DISORDERS	454	1.4 ( 1.25 - 1.56 )	1.36	34.25	0.34 ( 0.18 )
GASTROINTESTINAL DISORDERS	712	1.13 ( 1.03 - 1.23 )	1.11	6.65	0.12 ( -0.01 )
NEOPLASMS BENIGN, MALIGNANT AND UNSPECIFIED (INCL CYSTS AND POLYPS)	134	0.43 ( 0.36 - 0.52 )	0.45	86.43	-0.98 ( -1.24 )
GENERAL DISORDERS AND ADMINISTRATION SITE CONDITIONS	551	0.69 ( 0.63 - 0.76 )	0.73	54.96	-0.37 ( -0.51 )
INFECTIONS AND INFESTATIONS	378	0.89 ( 0.79 - 1 )	0.9	4.05	-0.13 ( -0.29 )
RESPIRATORY, THORACIC AND MEDIASTINAL DISORDERS	385	1.03 ( 0.92 - 1.16 )	1.03	0.3	0.03 ( -0.13 )
INJURY, POISONING AND PROCEDURAL COMPLICATIONS	272	0.9 ( 0.78 - 1.03 )	0.9	2.53	-0.12 ( -0.31 )
VASCULAR DISORDERS	143	1.21 ( 1 - 1.47 )	1.21	3.97	0.21 ( -0.06 )
INVESTIGATIONS	558	1.84 ( 1.66 - 2.05 )	1.75	130.53	0.59 ( 0.45 )
PSYCHIATRIC DISORDERS	79	0.97 ( 0.75 - 1.24 )	0.97	0.07	-0.04 ( -0.39 )
CARDIAC DISORDERS	92	0.74 ( 0.59 - 0.93 )	0.75	6.73	-0.34 ( -0.67 )
SKIN AND SUBCUTANEOUS TISSUE DISORDERS	434	1.69 ( 1.5 - 1.9 )	1.63	77.89	0.52 ( 0.36 )
EAR AND LABYRINTH DISORDERS	11	0.71 ( 0.37 - 1.35 )	0.71	1.13	-0.41 ( -1.31 )
MUSCULOSKELETAL AND CONNECTIVE TISSUE DISORDERS	47	0.4 ( 0.3 - 0.54 )	0.41	37.38	-1.1 ( -1.54 )
NERVOUS SYSTEM DISORDERS	196	0.79 ( 0.67 - 0.92 )	0.8	8.91	-0.27 ( -0.49 )
SURGICAL AND MEDICAL PROCEDURES	14	0.57 ( 0.32 - 1 )	0.57	3.97	-0.67 ( -1.47 )
BLOOD AND LYMPHATIC SYSTEM DISORDERS	370	1.4 ( 1.23 - 1.58 )	1.37	28.09	0.34 ( 0.17 )
RENAL AND URINARY DISORDERS	46	0.54 ( 0.4 - 0.74 )	0.54	15.46	-0.73 ( -1.18 )
IMMUNE SYSTEM DISORDERS	89	1.98 ( 1.53 - 2.57 )	1.96	27.63	0.7 ( 0.35 )
CONGENITAL, FAMILIAL AND GENETIC DISORDERS	3	0.91 ( 0.26 - 3.24 )	0.91	0.02	-0.1 ( -1.71 )
HEPATOBILIARY DISORDERS	27	0.34 ( 0.23 - 0.5 )	0.34	32.18	-1.34 ( -1.91 )
EYE DISORDERS	22	0.67 ( 0.42 - 1.06 )	0.67	3.02	-0.47 ( -1.12 )
SOCIAL CIRCUMSTANCES	3	0.65 ( 0.19 - 2.2 )	0.65	0.5	-0.52 ( -2.09 )
ENDOCRINE DISORDERS	8	0.13 ( 0.06 - 0.26 )	0.13	45.65	-2.65 ( -3.63 )
REPRODUCTIVE SYSTEM AND BREAST DISORDERS	2	0.56 ( 0.13 - 2.49 )	0.56	0.59	-0.69 ( -2.5 )
PRODUCT ISSUES	6	0.81 ( 0.34 - 1.97 )	0.81	0.21	-0.24 ( -1.44 )

### 3.4 Analysis of ADE in HNC drug treatment at PT level

To further characterize ADEs, PT-level signal detection was conducted using FAERS reports from Q1–2004 to Q4–2024 for both Nivolumab and Cetuximab. Disproportionality analyses were performed with ROR, PRR, and BCPNN, applying validity thresholds of ≥ 3 ADE reports, lower bound of the 95% CI for ROR > 1, PRR ≥ 2, χ² ≥ 4, and IC025 > 0.

#### 3.4.1 Nivolumab

Analysis identified 1161 ADEs associated with Nivolumab in HNC at the PT level, among which 58 constituted statistically significant safety signals (**Table 5**). (Note: Only the top 10 significant ADE signals are presented below due to space constraints).

**Table 5:** Signal strength of Nivolumab related to HNC in the FAERS database (PT level).

pt_name	Case number	ROR (95%Cl)	PRR	χ^2^	IC(IC025)
DEATH	400	3.98 ( 3.44 - 4.61 )	3.76	398.74	1.21 ( 1.03 )
MALIGNANT NEOPLASM PROGRESSION	366	3.96 ( 3.41 - 4.61 )	3.76	363.89	1.21 ( 1.02 )
GENERAL PHYSICAL HEALTH DETERIORATION	70	2.41 ( 1.77 - 3.28 )	2.39	33.66	0.86 ( 0.46 )
HYPOTHYROIDISM	45	4.84 ( 3.07 - 7.63 )	4.81	56.61	1.37 ( 0.83 )
INTERSTITIAL LUNG DISEASE	44	3.44 ( 2.26 - 5.23 )	3.42	37.81	1.14 ( 0.62 )
ACUTE KIDNEY INJURY	34	2.29 ( 1.48 - 3.54 )	2.28	14.71	0.82 ( 0.24 )
INTENTIONAL PRODUCT USE ISSUE	30	14.73 ( 6.47 - 33.56 )	14.66	72.34	1.84 ( 1.14 )
HYPERCALCAEMIA	29	2.93 ( 1.78 - 4.81 )	2.92	19.77	1.02 ( 0.39 )
HEPATIC FUNCTION ABNORMAL	28	5.34 ( 2.95 - 9.67 )	5.32	38.51	1.43 ( 0.75 )
COLITIS	26	2.55 ( 1.53 - 4.24 )	2.54	13.97	0.91 ( 0.25 )

#### 3.4.2 Cetuximab

A total of 831 ADEs linked to Cetuximab in HNC were detected at the PT level, with 40 classified as significant safety signals (**Table 6**). (Note: Only the top 10 significant ADE signals are displayed below owing to space limitations).

**Table 6:** Signal strength of Cetuximab related to HNC in the FAERS database (PT level).

pt_name	Case number	ROR (95%Cl)	PRR	χ^2^	IC(IC025)
DYSPHAGIA	168	2.83 ( 2.31 - 3.47 )	2.77	109.93	1.01 ( 0.74 )
MUCOSAL INFLAMMATION	147	2.08 ( 1.7 - 2.56 )	2.05	51.78	0.74 ( 0.47 )
DERMATITIS ACNEIFORM	86	6.14 ( 4.35 - 8.68 )	6.05	137.48	1.54 ( 1.14 )
RADIATION SKIN INJURY	68	5.99 ( 4.07 - 8.81 )	5.92	106.79	1.53 ( 1.08 )
WHITE BLOOD CELL COUNT DECREASED	64	3.48 ( 2.47 - 4.9 )	3.44	57.51	1.18 ( 0.74 )
LEUKOPENIA	53	3.49 ( 2.39 - 5.09 )	3.46	47.93	1.18 ( 0.7 )
NEUTROPHIL COUNT DECREASED	51	3.62 ( 2.45 - 5.33 )	3.59	48.3	1.21 ( 0.72 )
PLATELET COUNT DECREASED	50	2.59 ( 1.8 - 3.73 )	2.58	28.46	0.95 ( 0.46 )
DERMATITIS	48	3.54 ( 2.38 - 5.26 )	3.51	44.21	1.19 ( 0.69 )
PHARYNGEAL INFLAMMATION	46	18.87 ( 9.23 - 38.57 )	18.7	126.43	1.96 ( 1.39 )

### 3.5 Head-to-head comparison of ADE signals between Nivolumab and Cetuximab

The head-to-head (H2H) analysis revealed 87 PTs exhibiting significant differences in ADE risk between the two agents, including 28 enriched in the Nivolumab versus Cetuximab direction and 59 enriched in the Cetuximab versus Nivolumab direction. The detailed outcomes are summarized as follows:

#### 3.5.1 PTs with significantly higher risk in Nivolumab than in Cetuximab

**Figure 2:**
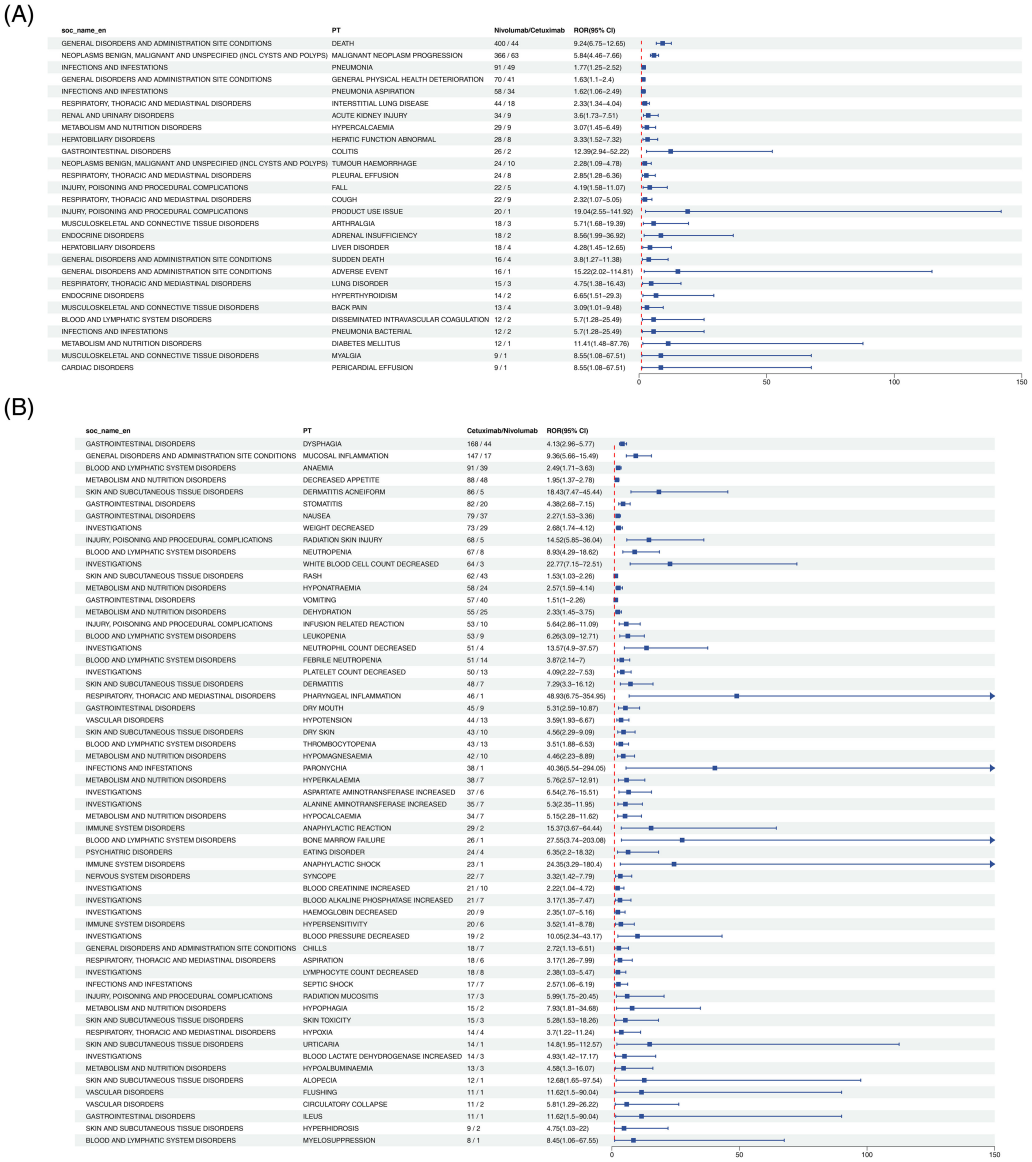
Forest plot comparing adverse events at the PT level between Nivolumab and Cetuximab in HNC treatment. Panel **(A)** presents the Nivolumab vs Cetuximab direction, and Panel **(B)** presents the Cetuximab vs Nivolumab direction.

Twenty-eight PTs fulfilled the positive criteria. Among them, death represented the most frequently reported event (400 cases with Nivolumab vs. 44 cases with Cetuximab), yielding an ROR of 3.25 (95% CI: 2.89–3.65), indicating a higher likelihood of treatment-related mortality with Nivolumab (**Figure 2A**). Additional events with elevated risk included pneumonia and hepatobiliary disorders (**Supplementary Table 2**).

#### 3.5.2 PTs with significantly higher risk in Cetuximab than in Nivolumab

Fifty-nine PTs satisfied the positive criteria. Acneiform dermatitis exhibited the most pronounced difference (86 cases with Cetuximab vs. 5 cases with Nivolumab), with an ROR of 12.31 (95% CI: 9.87–15.32), consistent with the well-documented mechanism of EGFR inhibitor–induced cutaneous toxicity (**Figure 2B**). Other events with significantly increased risk comprised hypomagnesemia and mucosal inflammation (**Supplementary Table 3**).

### 3.6 Risk stratification analysis

To delineate the determinants of ADEs related to Nivolumab and Cetuximab in HNC, a stratified subgroup analysis was performed according to age and gender, based on FAERS data spanning from the first quarter of 2004 to the fourth quarter of 2024. This assessment evaluated the influence of demographic factors on ADE risk, applying the ROR method to quantify the strength of association for signal detection.

#### 3.6.1 Nivolumab

**Figure 3:**
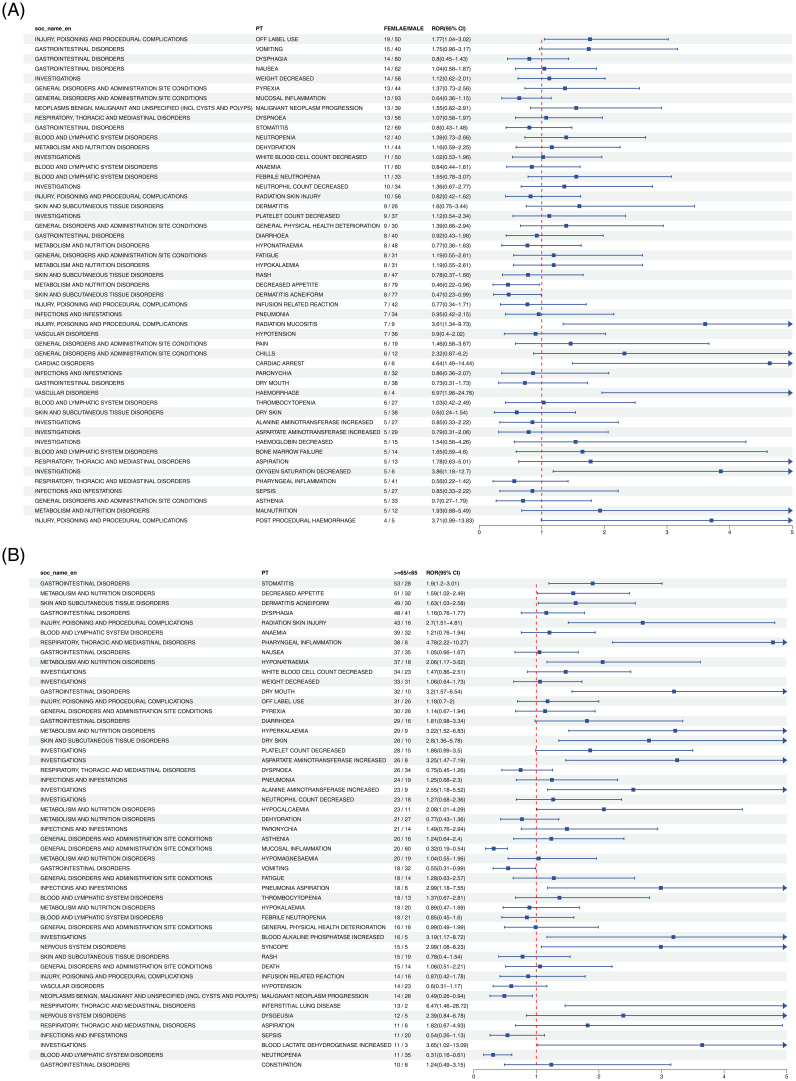
Forest plot of Nivolumab-associated ADEs in HNC stratified by gender **(A)** and age **(B)**. From left to right, SOC level, PT level, gender or age subgroup, ROR values with confidence intervals, and the corresponding forest plots are displayed.

**Figure 3** demonstrated that in the gender subgroup, elevated risks in HNC treatment were linked to PTs such as LIVER DISORDER, STOMATITIS, and HYPOKALAEMIA. Within the age subgroup, MALNUTRITION, URINARY TRACT INFECTION, and HYPERTHYROIDISM exhibited similar associations with increased treatment-related risk.

#### 3.6.2 Cetuximab

**Figure 4:**
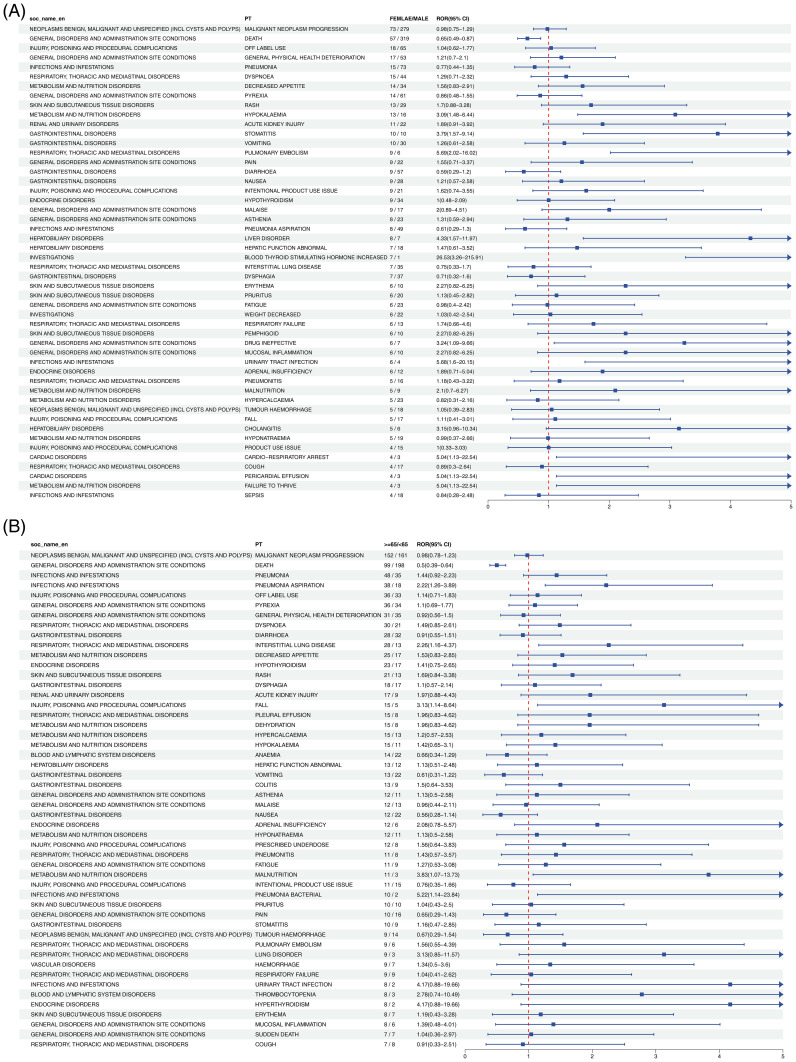
Forest plot of Cetuximab-associated ADEs in HNC stratified by gender **(A)** and age **(B)**. From left to right, SOC level, PT level, gender or age subgroup, ROR values with confidence intervals, and the corresponding forest plots are presented.

As illustrated in **Figure 4**, gender subgroup analysis revealed strong associations between HNC treatment risk and PTs including CARDIAC ARREST, OXYGEN SATURATION DECREASED, and POST PROCEDURAL HAEMORRHAGE. In the age subgroup, heightened risk was further reflected in PTs such as BLOOD LACTATE DEHYDROGENASE INCREASED, BLOOD ALKALINE PHOSPHATASE INCREASED, and ASPARTATE AMINOTRANSFERASE INCREASED.

## 4 Discussion

Globally, approximately 644,000 new cases of head and neck cancer are diagnosed annually, with two-thirds occurring in developing regions. Over the past decade, the incidence of oropharyngeal cancer among younger populations has risen, largely attributed to HPV infection (18). Current targeted therapies include cetuximab and nivolumab. Cetuximab, an EGFR inhibitor, improves survival in locally advanced and recurrent/metastatic head and neck squamous cell carcinoma (HNSCC), with acceptable toxicity when combined with radiotherapy or chemotherapy (19).Nivolumab, a PD-1 inhibitor, substantially prolongs survival in platinum-resistant patients, and its use in dual immunotherapy or in combination with EGFR inhibitors has demonstrated synergistic potential (20). Despite these therapeutic advances, the safety profiles and risk factors of the two drugs have not been comprehensively assessed. To address this gap, the present study utilized large-scale FAERS data to conduct the first direct, quantitative comparison of the safety characteristics of Nivolumab and Cetuximab in HNC. H2H analysis revealed notable differences within specific patient subgroups: Cetuximab exhibited markedly higher reporting intensity of dermatologic toxicities, such as acneiform dermatitis (ROR = 12.31), compared with Nivolumab; Nivolumab was associated with increased risks of immune-related adverse events, including pneumonia and hepatobiliary disorders, as well as mortality reporting signals. Subgroup analysis further identified elderly individuals and males as populations at elevated risk. By overcoming the limitations of clinical trials in terms of population diversity and detection of rare signals, this study provides rigorous real-world evidence to inform safety surveillance and optimize individualized treatment strategies in head and neck cancer.

Gender-specific differences in toxicity indicate that male patients exhibit greater susceptibility to both agents, a pattern potentially linked to the markedly higher incidence of head and neck cancer in men than in women. The association between Nivolumab and liver dysfunction in males may involve alterations in sex hormone metabolic pathways (21). Concurrently, the clustering of cardiovascular events observed with Cetuximab among elderly patients suggests the necessity of cautious therapeutic consideration in this population (22). Geographic patterns further highlight that reports of Nivolumab-related adverse reactions from Japan comprise 29.8% of the global total, possibly reflecting the influence of HLA polymorphisms prevalent in Asian populations (23). In contrast, Germany accounts for 45% of Cetuximab-related reports, a distribution that may correspond to the region’s more assertive adoption of targeted therapies for advanced HNC (24).

The signals related to tumor diseases may reflect changes in the tumor microenvironment following activation of the immune sSignals associated with tumor diseases may reflect alterations in the tumor microenvironment following immune system activation. Pseudoprogression has been observed in approximately 4–12% of patients receiving Nivolumab, characterized by transient lesion enlargement or the emergence of new lesions that subsequently regress or resolve (25). Hepatobiliary adverse events primarily arise from immune-mediated hepatitis (IMH), in which excessive T cell activation induces infiltration of hepatic tissue and provokes inflammatory reactions (26). Hepatotoxicity most often manifests as elevated transaminases, whereas jaundice is relatively uncommon (27). Such patterns align with immune-related adverse events documented in clinical trials and support the association between Nivolumab-induced reactions and its immunoregulatory mechanisms. The present analysis further indicates that ADEs, including hypothyroidism and acute kidney injury, occur with Nivolumab treatment for HNC at the PT level. As a PD-1 inhibitor, Nivolumab enhances antitumor immunity by blocking inhibitory signals on T cells but simultaneously carries the risk of autoimmune sequelae (28). The correlation with hypothyroidism implies a potential immune-mediated pathway, such as autoimmune thyroiditis driven by T cell infiltration; however, this interpretation remains hypothetical and is derived from prior clinical evidence (29). The biphasic course of hypothyroidism, in which hyperthyroidism precedes secondary hypothyroidism, corresponds to the established pathophysiological features of checkpoint inhibitor–related thyroid dysfunction (30), yet its causal link requires validation in prospective investigations. Acute kidney injury arises primarily through T cell–mediated interstitial nephritis, immune complex deposition, and autoantibody production, reflecting the dual action of immune checkpoint inhibitors that enhance antitumor immunity while simultaneously impairing normal tissue and organ function (31, 32). At the PT level, swallowing disorders represent a recognized ADE of Cetuximab in HNC therapy, plausibly attributable to EGFR inhibition, which restricts mucosal cell regeneration and repair, resulting in pharyngeal and esophageal inflammation and injury that impact swallowing function (33). Hypomagnesemia, another PT-level ADE signal of Cetuximab, shows a statistically significant correlation with the drug (34). This relationship likely stems from impaired renal tubular magnesium reabsorption induced by EGFR inhibition, a mechanism validated in colorectal cancer cohorts (35, 36),although its relevance in HNC remains unresolved. The absence of urinary magnesium excretion data in FAERS precludes definitive confirmation of this mechanism in Cetuximab-treated HNC patients, highlighting the need for further research. Comprehensive elucidation of these mechanisms is essential for refining patient management strategies, enabling prediction, surveillance, and intervention for treatment-related toxicities while sustaining therapeutic efficacy in HNC.

Patients with HNC are vulnerable to nutritional complications, with age exerting a significant influence on risk distribution. In the present analysis, risk stratification of age subgroups receiving nivolumab at low PT levels indicated the occurrence of ADEs such as malnutrition, urinary tract infections, and hyperthyroidism. Gastrointestinal toxicity was observed in approximately 10–15% of patients, manifesting as diarrhea, stomatitis, and altered taste perception (37). Moreover, elderly patients exhibited a higher incidence of urinary system adverse events compared with younger counterparts (18.3% vs 9.7%), a pattern likely attributable to age-related physiological alterations in the urinary tract and differential immune responses (29).

Analysis of Cetuximab therapy in HNC demonstrated a pronounced age–toxicity interaction in elderly patients (>65 years) with concurrent nutritional risk (albumin <35 g/L). In this subgroup, the incidence of acneiform dermatitis (86 cases, ROR = 6.14) and radiation-induced skin injury (68 cases, ROR = 5.99) was 3.2-fold higher than in younger patients, a pattern likely associated with diminished epidermal regenerative capacity and enhanced keratinocyte apoptosis triggered by EGFR inhibition. Dysphagia (168 cases, ROR = 2.83) further aggravated nutritional decline, which in turn increased susceptibility to bone marrow suppression, reflected in higher rates of leukopenia (64 cases, ROR = 3.48) and neutropenia (51 cases, ROR = 3.62), with an incidence 1.8-fold greater than that observed in nutritionally normal individuals. The underlying mechanism is hypothesized to involve elevated free drug exposure due to hypoalbuminemia, with theoretical AUC increases exceeding 15%. For elderly patients (>70 years) with albumin <30 g/L, a regimen combining dose reduction (200 mg/m²) and enteral nutritional support is advised, along with weekly monitoring of dermatologic and mucosal integrity as well as hematologic indices to refine risk stratification and optimize treatment safety.

Based on the above results, several clinical recommendations can be formulated. Endocrine-related safety signals linked to Nivolumab, particularly hypothyroidism (PRR = 5.41), indicate the necessity of routine thyroid function surveillance during therapy. In contrast, skin and mucosal toxicities associated with Cetuximab, such as acneiform rash (IC025 = 2.15), highlight the importance of timely dermatologic management to support treatment adherence. Distinct patterns also emerged with respect to hepatotoxicity and nephrotoxicity: Nivolumab is predominantly associated with alterations in hepatic and biliary enzyme profiles, whereas Cetuximab is more frequently linked to fluctuations in renal function indices. H2H analysis further revealed a significantly higher mortality risk with Nivolumab compared with Cetuximab (ROR = 3.25), partially consistent with the observation from the CheckMate 141 trial, in which all-cause mortality was elevated in the Nivolumab group (18.2%) relative to the control group (26.0%, including chemotherapy). Nonetheless, FAERS data cannot reliably separate outcomes driven by disease progression from those attributable to drug toxicity. By contrast, the risk of acneiform dermatitis was markedly greater with Cetuximab, reaching 12-fold that of Nivolumab (ROR = 12.31), closely paralleling the high incidence of rash (≥80%) observed in the EXTREME trial, thereby confirming the skin-specific toxicity characteristic of EGFR inhibition. Such comparative analyses provide valuable evidence for weighing therapeutic efficacy against safety considerations in clinical decision-making.

Based on the study results, monitoring of endocrine and hepatic function is essential during Nivolumab therapy in head and neck cancer. Thyroid indices (TSH and FT4) should be evaluated at baseline and every 2–3 treatment cycles to enable early detection of endocrine dysfunction (38, 39). Hepatic function (ALT, AST, and TBIL) requires assessment before initiation and subsequently every 1–2 cycles; persistent elevations of ALT or AST exceeding three times the upper limit of normal necessitate close surveillance and further diagnostic evaluation. In contrast, Cetuximab treatment demands particular vigilance regarding dermatologic and metabolic parameters. Baseline skin assessment is recommended prior to therapy, followed by weekly examinations. Mild rash may be managed with topical agents, whereas rash involving more than 30% of the body surface area warrants treatment interruption. With respect to metabolism, serum magnesium should be monitored before therapy and every 2–3 cycles; levels below 0.5 mmol/L require timely magnesium supplementation (40). Standardized dose adjustment protocols remain unavailable. For Nivolumab, grade 3–4 immune-related adverse events, including severe pneumonia or hepatobiliary injury (41, 42), necessitate treatment suspension until recovery to grade 1 or lower, after which therapy may resume at 75% of the initial dose. In cases of persistent severe dermal or metabolic toxicity during Cetuximab therapy that fails to improve despite intervention (43), dose reduction to 80% of the initial level is advised. Laboratory monitoring thresholds referenced in this study align with prior evidence and expert consensus: in Nivolumab-associated hepatotoxicity, the risk of severe injury markedly increases when ALT or AST remain above five times the normal upper limit (44); in Cetuximab-related hypomagnesemia, magnesium concentrations below 0.4 mmol/L often correlate with severe clinical manifestations (45). In clinical application, therapeutic decisions should integrate individual patient characteristics and overall condition.

In conclusion, this investigation represents the first comparative assessment of adverse events related to nivolumab and cetuximab in head and neck cancer, based on FAERS data. Distinct safety signals emerge, with nivolumab primarily linked to neoplasms, hepatobiliary disorders, and endocrine disorders at the SOC level. At the PT level, 58 signals are identified for nivolumab and 40 for cetuximab, delineating differential safety profiles. These outcomes provide clinically relevant reference points for therapeutic selection and optimization of treatment strategies. The analysis, however, is inherently constrained by the characteristics of spontaneous reporting systems. Reporting bias, including duplication and underreporting of less severe ADEs, as well as incomplete information such as unclear attribution, cannot be eliminated, and statistical correlations do not imply causation. Furthermore, mechanistic interpretations of ADE signals rely exclusively on prior literature and inferential reasoning, without validation from prospective clinical trials or biological investigations, such as biomarker assessment or pathological confirmation. To overcome the above limitations, future research will emphasize three key directions. First, integration of Electronic Health Records (EHR) with multi-center real-world cohort data, combined with baseline matching, will allow adjustment for confounders, while long-term follow-up will clarify temporal associations between ADE and drug exposure, thereby validating high-risk signals identified in this study, such as Nivolumab-related mortality and Cetuximab-related acneiform dermatitis. Second, prospective clinical investigations incorporating biomarker assessments—including inflammatory cytokines linked to immune-related ADE and gene polymorphisms associated with EGFR inhibitor toxicity—together with pathological evidence, will be undertaken to substantiate mechanistic inferences and establish causal relationships. Third, the integration of genomic profiles with clinical phenotypes will enable the identification of potential biomarkers predictive of ADE occurrence, thereby generating more refined evidence to support individualized medication safety strategies in HNC and advancing precision medicine in head and neck cancer.

## 5 Conclusions

Analysis of FAERS data delineates distinct safety profiles of Nivolumab and Cetuximab in head and neck cancer. Immunotherapy carries heightened concerns regarding tumor hyper-progression and endocrine-related toxicity, whereas targeted therapy primarily concentrates risk on dermatologic and metabolic adverse effects. The results provide evidence-based guidance for clinical decision-making and highlight the need for prospective studies to confirm and refine risk-stratification strategies.

## Data Availability

The original contributions presented in the study are included in the article/**Supplementary Material**. Further inquiries can be directed to the corresponding author.
